# Crystal structure, Hirshfeld surface analysis, inter­action energy and DFT calculations and energy frameworks of methyl 6-chloro-1-methyl-2-oxo-1,2-di­hydro­quinoline-4-carboxyl­ate

**DOI:** 10.1107/S2056989022002912

**Published:** 2022-03-22

**Authors:** Yassir Filali Baba, Sonia Hayani, Samira Dalbouha, Tuncer Hökelek, Fouad Ouazzani Chahdi, Joel T. Mague, Youssef Kandri Rodi, Nada Kheira Sebbar, El Mokhtar Essassi

**Affiliations:** a Laboratory of Applied Organic Chemistry, Faculty of Science and Technology, Sidi Mohammed Ben Abdullah University, Route d’Immouzzer, BP 2202, Fez, Morocco; b Laboratory of Spectroscopy, Molecular Modeling, Materials, Nanomaterials, Water and Environment, CERNE2D, Faculty of Sciences-Rabat, Mohammed V University, Av. Ibn Battouta, BP 1014 Rabat, Morocco; c Research team: Materials and Environmental Applications, Laboratory of Applied Chemistry and Environment, Department of Chemistry, Faculty of Sciences-Agadir, Ibn Zohr University, BP 8106 Agadir, Morocco; d Department of Physics, Hacettepe University, 06800 Beytepe, Ankara, Turkey; e Department of Chemistry, Tulane University, New Orleans, LA 70118, USA; f Laboratory of Applied Chemistry and Environment, Applied Bioorganic Chemistry Team, Faculty of Science, Ibn Zohr University, Agadir, Morocco; g Laboratory of Heterocyclic Organic Chemistry, Pharmacochemistry Competence Center, Drug Science Research Center, Faculty of Sciences, Mohammed V University of Rabat, Rabat, Morocco

**Keywords:** crystal structure, hydrogen-bonding, π-stacking, di­hydro­quinoline

## Abstract

In the title compound, C_12_H_10_ClNO_3_, the di­hydro­quinoline moiety is not planar with a dihedral angle between the two ring planes of 1.61 (6)°. An intra­molecular C—H⋯O hydrogen bond helps to establish the rotational orientation of the carboxyl group. In the crystal, sheets of mol­ecules parallel to (10  ) are generated by C—H⋯O and C—H⋯Cl hydrogen bonds, and are stacked through slipped π-stacking inter­actions between inversion-related di­hydro­quinoline units. A Hirshfeld surface analysis of the crystal structure indicates that the most important contributions for the crystal packing are from H⋯H (34.2%), H⋯O/O⋯H (19.9%), H⋯Cl/Cl⋯H (12.8%), H⋯C/C⋯H (10.3%) and C⋯C (9.7%) inter­actions. Computational chemistry indicates that in the crystal, the C—H⋯Cl hydrogen-bond energy is −37.4 kJ mol^−1^, while the C—H⋯O hydrogen-bond energies are −45.4 and −29.2 kJ mol^−1^. An evaluation of the electrostatic, dispersion and total energy frameworks revealed that the stabilization is dominated *via* the dispersion energy contribution. Density functional theory (DFT) optimized structures at the B3LYP/6–311 G(d,p) level are compared with the experimentally determined mol­ecular structure in the solid state, and the HOMO—LUMO behaviour was elucidated to determine the energy gap.

## Abstract 2

In the title compound, the di­hydro­quinoline moiety is not quite planar and an intra­molecular C—H⋯O hydrogen bond helps to establish the rotational orientation of the carboxyl group. In the crystal, sheets of mol­ecules parallel to (10 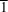 ) are generated by C—H⋯O and C—H⋯Cl hydrogen bonds.

## Chemical context

Over the past few decades, heterocyclic chemistry has received increasing inter­est because of the pharmacological importance of the majority of heterocyclic compounds, especially N-containing heterocycles such as quinoline derivatives (Filali Baba *et al.*, 2019 ▸; Hayani *et al.*, 2021 ▸). Quinoline derivatives possess numerous biological properties, including anti­microbial (Katoh *et al.*, 2004 ▸; Abdel-Wahab *et al.*, 2012 ▸), anti-inflammatory (Leatham *et al.*, 1983 ▸), anti­hypertensive (Muruganantham *et al.*, 2004 ▸), anti­biotic (Mahamoud *et al.*, 2006 ▸), anti-HIV (Wilson *et al.*, 1992 ▸; Strekowski *et al.*, 1991 ▸) and corrosion-inhibitive activities (Filali Baba *et al.*, 2016*a* ▸,*b* ▸). They are also considered to be important scaffolds for the development of new mol­ecules of pharmaceutical inter­est (Filali Baba *et al.*, 2020 ▸; Bouzian *et al.*, 2018 ▸). 
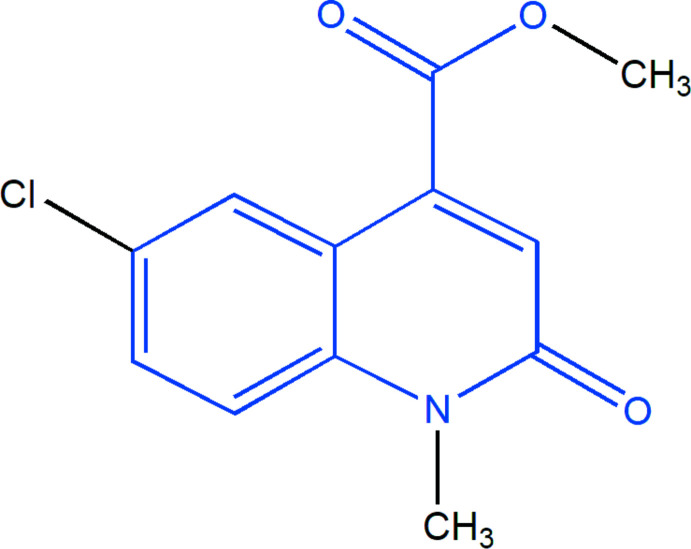


In a continuation of our research work devoted to the study of O- and N-alkyl­ation reactions involving quinoline derivatives, we report here the synthesis and crystal structure of methyl 6-chloro-1-methyl-2-oxo-1,2-di­hydro­quinoline-4-carboxyl­ate obtained by the alkyl­ation reaction of 6-chloro-2-oxo-1,2-di­hydro­quinoline-4-carb­oxy­lic acid with an excess of methyl iodide as an alkyl­ating reagent in phase transfer catalysis (PTC). The mol­ecular and crystal structure as well as the Hirshfeld surface analysis of the title compound are reported. The results obtained using density functional theory (DFT) calculations, performed at the B3LYP/6-311G(d,p) level, are compared with the experimental results determined from the mol­ecular and crystal structures in the solid state of the title compound, (I).

## Structural commentary

**Figure 1:**
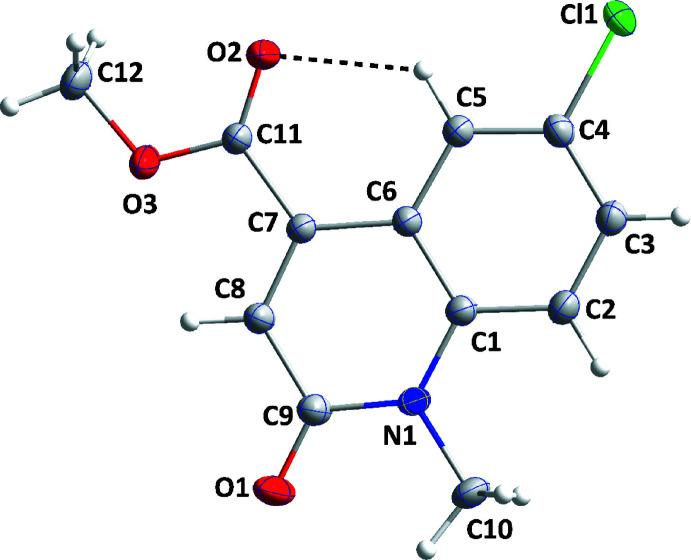
The title mol­ecule with labelling scheme and displacement ellipsoids drawn at the 50% probability level. The intra­molecular C—H⋯O hydrogen bond is depicted by a dashed line.

The bicyclic mol­ecular core is not planar as there is a dihedral angle of 1.61 (6)° between the mean planes of its constituent rings. The dihedral angle between the mean plane of the (C1/N1/C6–C9) ring and the plane defined by atoms C7, C11, O2 and O3 is 4.08 (8)° with the near coplanarity of the carboxyl group and the heterocyclic ring being caused, in part, by the intra­molecular C5—H5⋯O2 hydrogen bond (Table 1 ▸, Fig. 1 ▸).

## Supra­molecular features

**Figure 2:**
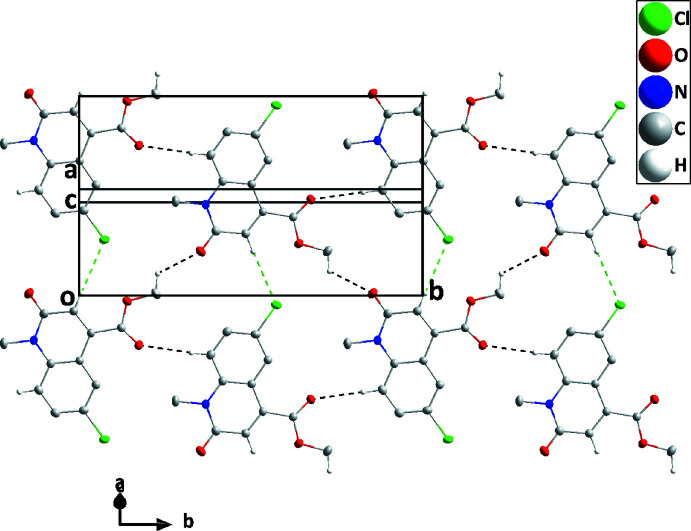
A portion of one layer projected on (10 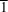 ) with C—H⋯O and C—H⋯Cl hydrogen bonds depicted, respectively, by black and green dashed lines.

**Figure 3:**
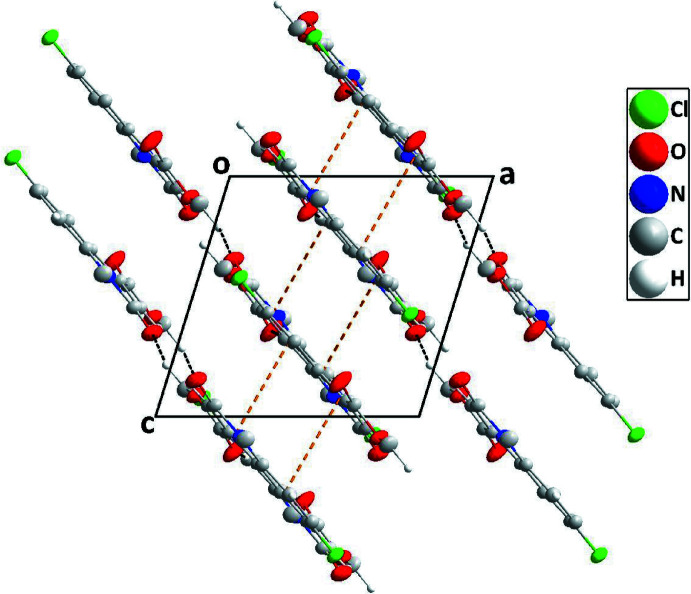
Packing of mol­ecules viewed along [010] with slipped π-stacking inter­actions depicted by orange dashed lines.

In the crystal, C2—H2⋯O2^iii^ hydrogen bonds (Table 1 ▸) form ribbons of mol­ecules extending along [010], which are further linked into sheets parallel to (10 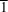 ) by C12—H12*C*⋯O1^ii^ and weak C8—H8⋯Cl1^iv^ (H⋯Cl is 0.11 Å less than the sum of the van der Waals radii) hydrogen bonds (Table 1 ▸, Fig. 2 ▸). The sheets are stacked along the direction of the normal to (10 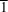 ) by slipped π-stacking inter­actions between inversion-related di­hydro­quinoline moieties [centroid⋯centroid distance = 3.7140 (7) Å, dihedral angle = 1.61 (6)°, slippage = 1.63 Å] (Fig. 3 ▸).

## Hirshfeld surface analysis

**Figure 4:**
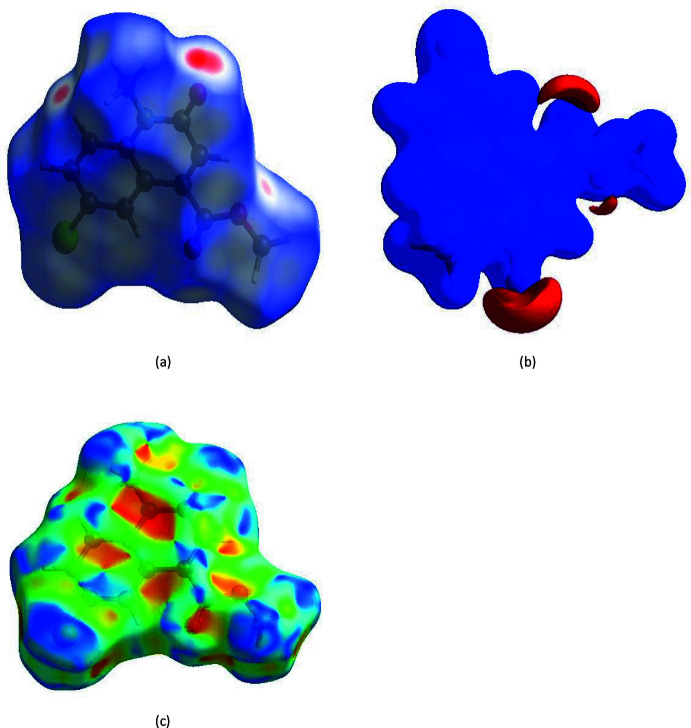
(*a*) View of the three-dimensional Hirshfeld surface of the title compound, plotted over *d*_norm_ in the range of −0.2172 to 0.9151 a.u.; (*b*) view of the three-dimensional Hirshfeld surface of the title compound plotted over electrostatic potential energy in the range −0.0500 to 0.0500 a.u. using the STO-3 G basis set at the Hartree–Fock level of theory; (*c*) Hirshfeld surface of the title compound plotted over shape-index.

**Figure 5:**
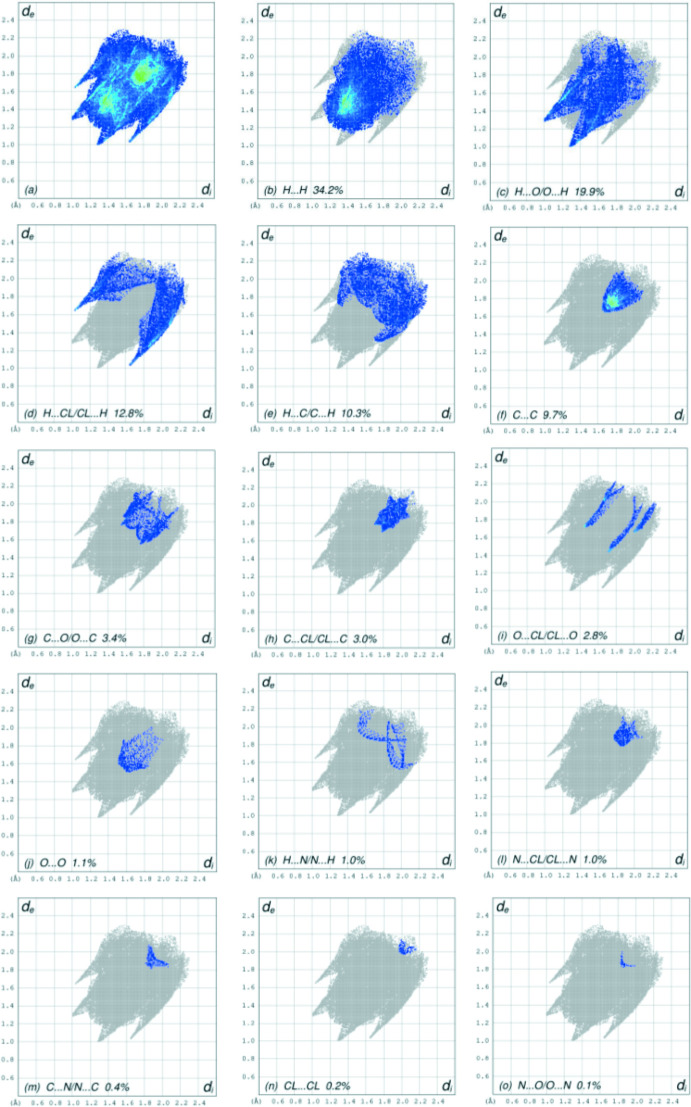
The full two-dimensional fingerprint plots for the title compound, showing (*a*) all inter­actions, and delineated into (*b*) H⋯H, (*c*) H⋯O/O⋯H, (*d*) H⋯Cl/Cl⋯H, (*e*) H⋯C/C⋯H, (*f*) C⋯C, (*g*) C⋯O/O⋯C, (*h*) C⋯Cl/Cl⋯C, (i) O⋯Cl/Cl⋯O, (*j*) O⋯O, (*k*) H⋯N/N⋯H, (*l*) N⋯Cl/Cl⋯N, (*m*) C⋯N/N⋯C, (*n*) Cl⋯Cl and (*o*) N⋯O/O⋯N inter­actions. The *d*_i_ and *d*_e_ values are the closest inter­nal and external distances (in Å) from given points on the Hirshfeld surface.

In order to visualize the inter­molecular inter­actions in the crystal of (I), a Hirshfeld surface (HS) analysis (Hirshfeld, 1977 ▸) was carried out by using *CrystalExplorer17.5* (Turner *et al.*, 2017 ▸). In the HS plotted over *d*_norm_ (Fig. 4 ▸ *a*), the white surface indicates contacts with distances equal to the sum of van der Waals radii, and the red and blue colours indicate distances shorter (in close contact) or longer (distinct contact) than the van der Waals radii (Venkatesan *et al.*, 2016 ▸). Selected contacts are given in Table 2 ▸. The bright-red spots indicate their roles as the respective donors and/or acceptors; they also appear as blue and red regions corresponding to positive and negative potentials on the HS mapped over electrostatic potential (Spackman *et al.*, 2008 ▸; Jayatilaka *et al.*, 2005 ▸) shown in Fig. 4 ▸ *b*. The blue regions indicate a positive electrostatic potential (hydrogen-bond donors), while the red regions indicate a negative electrostatic potential (hydrogen-bond acceptors). The shape-index of the HS is a tool to visualize π–π stacking by the presence of adjacent red and blue triangles: if there are no adjacent red and/or blue triangles, then there are no π–π inter­actions. Fig. 4 ▸ *c* clearly suggests that there are π–π inter­actions in (I). The overall two-dimensional fingerprint plot is shown in Fig. 5 ▸ *a*, and those delineated into H⋯H, H⋯O/O⋯H, H⋯Cl/Cl⋯H, H⋯C/C⋯H, C⋯C, C⋯O/O⋯C, C⋯Cl/Cl⋯C, O⋯Cl/Cl⋯O, O·· O, H⋯N/N⋯H, N⋯Cl/Cl⋯N, C⋯N/N⋯C, Cl⋯Cl and N⋯O/O⋯N contacts (McKinnon *et al.*, 2007 ▸) are illustrated in Fig. 5 ▸ *b*–*o*, respectively, together with their relative contributions to the Hirshfeld surface. The most important inter­action is H⋯H, contributing 34.2% to the overall crystal packing, which is reflected in Fig. 5 ▸ *b* as widely scattered points of high density due to the large hydrogen content of the mol­ecule with the tip at *d*_e_ = *d*_i_ = 1.24 Å. The pair of the scattered points of spikes in the fingerprint plot delineated into H⋯O/O⋯H contacts, Fig. 5 ▸ *c*, with a 19.9% contribution to the HS has a distribution of points with the tips at *d*_e_ + *d*_i_ = 2.28 Å. The H⋯Cl/Cl⋯H contacts, Fig. 5 ▸ *d*, with a 12.8% contribution to the HS have a symmetric distribution of points with the tips at *d*_e_ + *d*_i_ = 2.68 Å. In the absence of C—H⋯π inter­actions, the pair of characteristic wings in the fingerprint plot delineated into H⋯C/C⋯H contacts, Fig. 5 ▸ *e*, with a 10.3% contribution to the HS has the tips at *d*_e_ + *d*_i_ = 3.01 Å. The C⋯C contacts, Fig. 5 ▸ *f*, with a 9.7% contribution to the HS have a bullet-shaped distribution of points with the tip at *d*_e_ = *d*_i_ = 1.67 Å. The C⋯O/O⋯C contacts, Fig. 5 ▸ *g*, with a 3.4% contribution to the HS have the tips at *d*_e_ + *d*_i_ = 3.32 Å and *d*_e_ + *d*_i_ = 3.45 Å for sharp and tiny distributions of points. The symmetric distribution of points of the C⋯Cl/Cl⋯C contacts, Fig. 5 ▸ *h*, with a 3.0% contribution to the HS appear as scattered points with a tiny pair of spikes with the tips at *d*_e_ + *d*_i_ = 3.48 Å. Finally, the contributions of the remaining O⋯Cl/Cl⋯O, O⋯O, H⋯N/N⋯H, N⋯Cl/Cl⋯N, C⋯N/N⋯C, Cl⋯Cl and N⋯O/O⋯N contacts (Fig. 5 ▸ *i*–*o*) are smaller than 3.0% to the HS with low densities of points.

**Figure 6:**
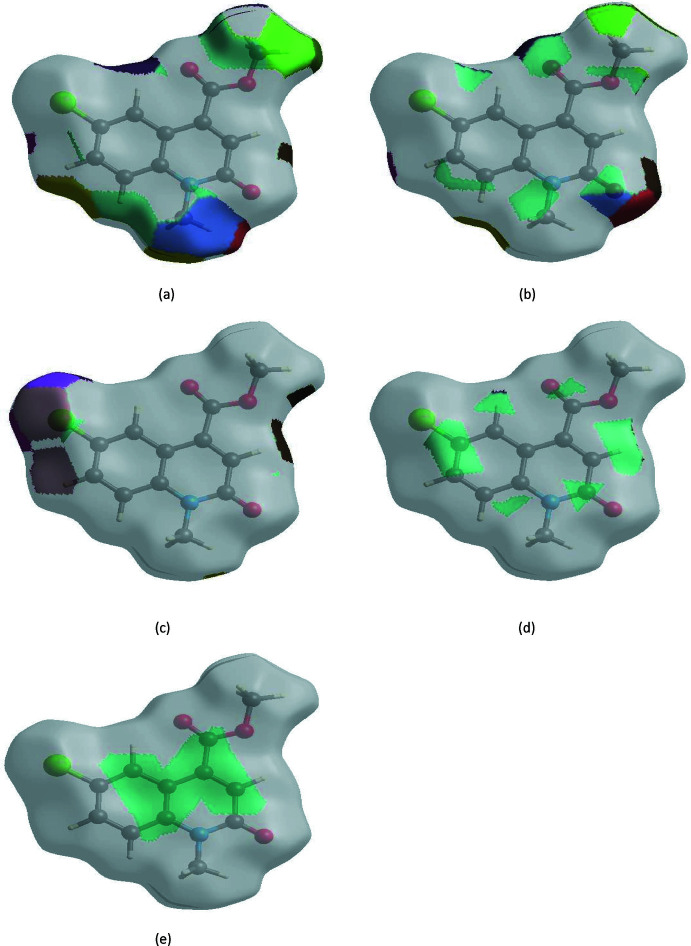
Hirshfeld surface representations with the function *d*_norm_ plotted onto the surface for (*a*) H⋯H, (*b*) H⋯O/O⋯H, (*c*) H⋯Cl/Cl⋯H, (*d*) H⋯C/C⋯H and (*e*) C⋯C inter­actions.

The Hirshfeld surface representations with the function *d*_norm_ plotted onto the surface are shown for the H⋯H, H⋯O/O⋯H, H⋯Cl/Cl⋯H, H⋯C/C⋯H and C⋯C inter­actions in Fig. 6 ▸ *a*–*e*, respectively.

The Hirshfeld surface analysis confirms the importance of H-atom contacts in establishing the packing. The large number of H⋯H, H⋯O/O⋯H, H⋯Cl/Cl⋯H and H⋯C/C⋯H inter­actions suggest that van der Waals inter­actions play the major role in the crystal packing (Hathwar *et al.*, 2015 ▸).

## Inter­action energy calculations

The inter­molecular inter­action energies were calculated using the CE–B3LYP/6–31G(d,p) energy model available in *CrystalExplorer17.5* (Turner *et al.*, 2017 ▸), where a cluster of mol­ecules would be needed by applying crystallographic symmetry operations with respect to a selected central mol­ecule within the radius of 3.8 Å by default (Turner *et al.*, 2014 ▸). The total inter­molecular energy (*E*_tot_) is the sum of electrostatic (*E*_ele_), polarization (*E*_pol_), dispersion (*E*_dis_) and exchange-repulsion (*E*_rep_) energies (Turner *et al.*, 2015 ▸) with scale factors of 1.057, 0.740, 0.871 and 0.618, respectively (Mackenzie *et al.*, 2017 ▸). Hydrogen-bonding inter­action energies (in kJ mol^−1^) were calculated to be −10.4 (*E*_ele_), −1.6 (*E*_pol_), −51.9 (*E*_dis_), 32.4 (*E*_rep_) and −37.4 (*E*_tot_) [for the C8—H8⋯Cl1^iv^ hydrogen-bonding inter­action], −0.9 (*E*_ele_), −2.8 (*E*_pol_), −84.0 (*E*_dis_), 49.9 (*E*_rep_) and −45.4 (*E*_tot_) (for C2—H2⋯O2^iii^) and −6.0 (*E*_ele_), −4.1 (*E*_pol_), −37.0 (*E*_dis_), 20.2 (*E*_rep_) and −29.2 (*E*_tot_) (for C12—H12*C*⋯O1^ii^).

## Energy frameworks

**Figure 7:**
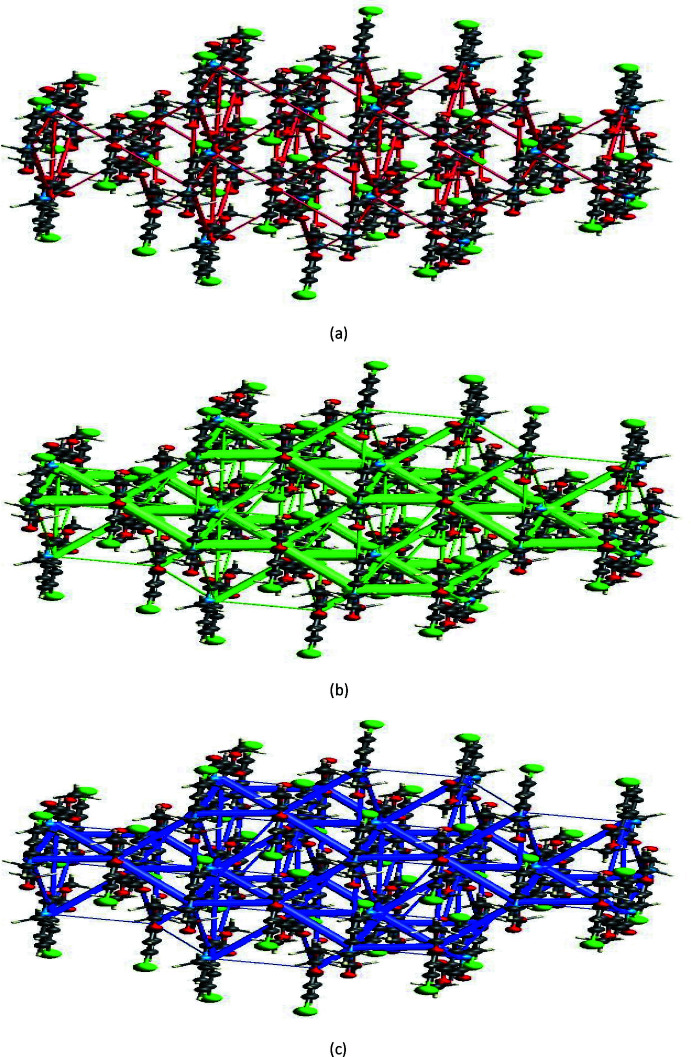
Energy frameworks of (I).

Energy frameworks combine the calculation of inter­molecular inter­action energies with a graphical representation of their magnitude (Turner *et al.*, 2015 ▸). Energies between mol­ecular pairs are represented as cylinders joining the centroids of pairs of mol­ecules with the cylinder radius proportional to the relative strength of the corresponding inter­action energy. Energy frameworks were constructed for *E*_ele_ (red cylinders), *E*_dis_ (green cylinders) and *E*_tot_ (blue cylinders) (Fig. 7 ▸ *a*–*c*). The evaluation of the electrostatic, dispersion and total energy frameworks indicates that the stabilization is dominated by the dispersion energy contributions in (I).

## DFT calculations

The geometrical parameters and energies of (I) in the gas phase were computed *via* density functional theory (DFT) using the standard B3LYP functional and 6–311G(d,p) basis-set calculations (Becke, 1993 ▸) as implemented in *GAUSSIAN 09* (Frisch *et al.*, 2009 ▸), see Table 3 ▸. The theoretical bond lengths and angles are in good agreement with those based on the X-ray analysis. However, a few differences exists in case of some dihedral angles (N1—C1—C6—C7; C10—N1—C1—C6; O1—C9—N1—C1; O2—C11—C7—C6; O3—C11—C7—C6; C12—O3—C11—C7), because in the DFT calculations there is only one mol­ecule treated in the gas phase whereas in the solid state several mol­ecules inter­act by hydrogen-bonding inter­actions (Fig. 2 ▸, Table 1 ▸). The torsion angles show that the conformation of the mol­ecule in the gas phase has *C*_1_ symmetry.

The infrared spectrum of (I) on basis of the B3LYP/6-311G calculation is shown in the supporting information. All harmonic frequencies are positive, demonstrating the minimal signature of (I). The spectrum mainly constitutes 75 vibration modes. The CH_3_ torsion appears in the 17–119 cm^−1^ region, the ν C=C stretching mode is at 1363 cm^−1^, the vibrations of the aromatic N—CH_3_ appear at 1091 cm^−1^, and the O—CH_3_ and the C—Cl stretching bands are observed, respectively, at 1033 cm^−1^ and 1124 cm^−1^. The C—H stretch of the CH_3_ group appears at 3182 cm^−1^, however the aromatic C—H stretches appear in the 3208-3256 cm^−1^ region. The bending of CH_3_ appear between 1528 cm^−1^ and 1556 cm^−1^, Finally, the band positions of the bending of the HCC, HCN and HCO groups are respectively at 1169 cm^−1^, 1119 cm^−1^ and 1204 cm^−1^.

**Figure 8:**
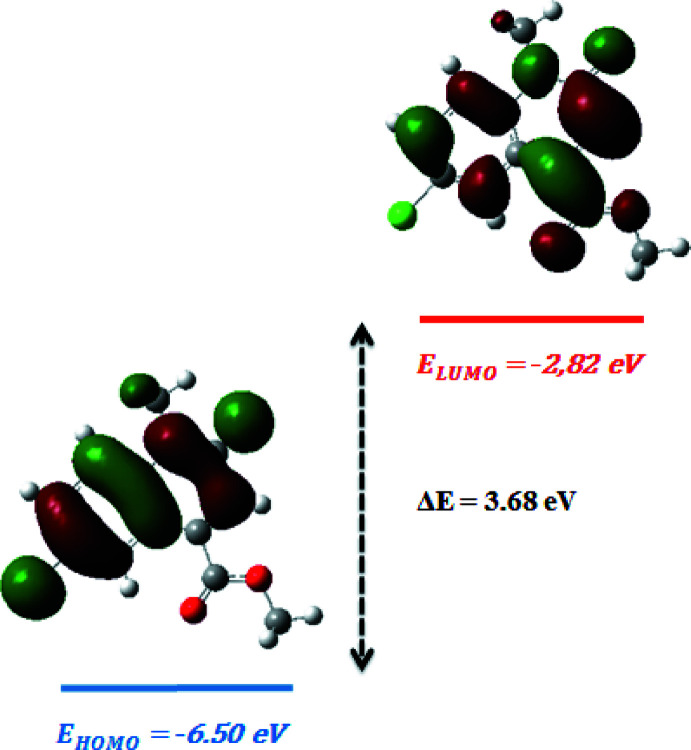
The energy band gap of (I).

The HOMO and LUMO energies are predicted with the B3LYP method in combination of basis sets 6-31G(d,p). This mol­ecule contains 65 occupied mol­ecular orbitals and 309 unoccupied virtual mol­ecular orbitals. The frontier mol­ecular orbitals are shown in Fig. 8 ▸. The positive phase is shown in red and the negative phase is shown in green. The HOMO-LUMO energy gap of (I) reflects the chemical activity and was calculated by the DFT/B3LYP/6-31G(d,p) method (Table 4 ▸). The high value of the energy gap (3.68 eV) implies a high electronic stability and low reactivity. In general, low values mean that it will be easier to remove an electron from the HOMO orbital towards the LUMO orbital.

## Mol­ecular electrostatic potential (MESP) analysis

**Figure 9:**
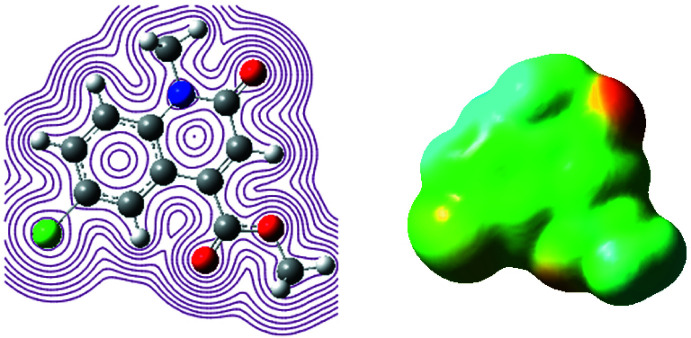
Contour surface of the electrostatic potential of (I).

The study of MESP is a useful tool in the investigation of the mol­ecular structure with its relation to physico-chemical properties. The MESP analysis of (I) was performed with the functional B3LYP and the basis set 6-311G (d,P). The different values of the electrostatic potential are represented by different colours (Seminario, 1996 ▸; Murray & Sen, 1996 ▸) such that red represents the region of the most negative electrostatic potential (electrophilic sites), blue represents the region of the most positive electrostatic potential (the nucleophilic reactivity) and green represents the region of zero potential. The potential increases in the following order: red < orange < yellow < green < blue. Fig. 9 ▸ reveals that the negative potential sites are on oxygen and chlorine atoms, as well as the positive potential site is around hydrogen atoms. From these results, we can deduce that the H atoms show the strongest attraction and the oxygen and chlorine atoms show the strongest repulsion in the density curve. The H atom of the meth­oxy and amine group has a higher positive value than the other H atoms.

## Database survey

A search of the Cambridge Crystallographic Database (updated to Dec. 31, 2021; Groom *et al.*, 2016 ▸) using the fragment shown in the scheme below yielded 20 hits of which 16 contained an ester group attached to C7 (the remainder contained an alkyl group at this position) and, of these, only two, ROKCIG (Filali Baba *et al.*, 2019 ▸) and REYREV (Filali Baba *et al.*, 2018 ▸) contain a halogen atom attached to the aromatic ring. The former is more closely related to the title mol­ecule by having an ethyl group attached to nitro­gen and also in the ester substituent. In contrast to the title mol­ecule, that in ROKCIG forms inversion dimers through C—H⋯O hydrogen bonds (rather than ribbons), which are connected into layers approximately parallel to (10 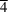 ), but there are no C—H⋯Cl hydrogen bonds or π-stacking inter­actions. In the non-halogenated analog of ROKCIG (ROKCOM; Filali Baba *et al.*, 2019 ▸) C—H⋯O hydrogen bonds form ribbons of mol­ecules along [001], which are connected by weak π-stacking inter­actions. 
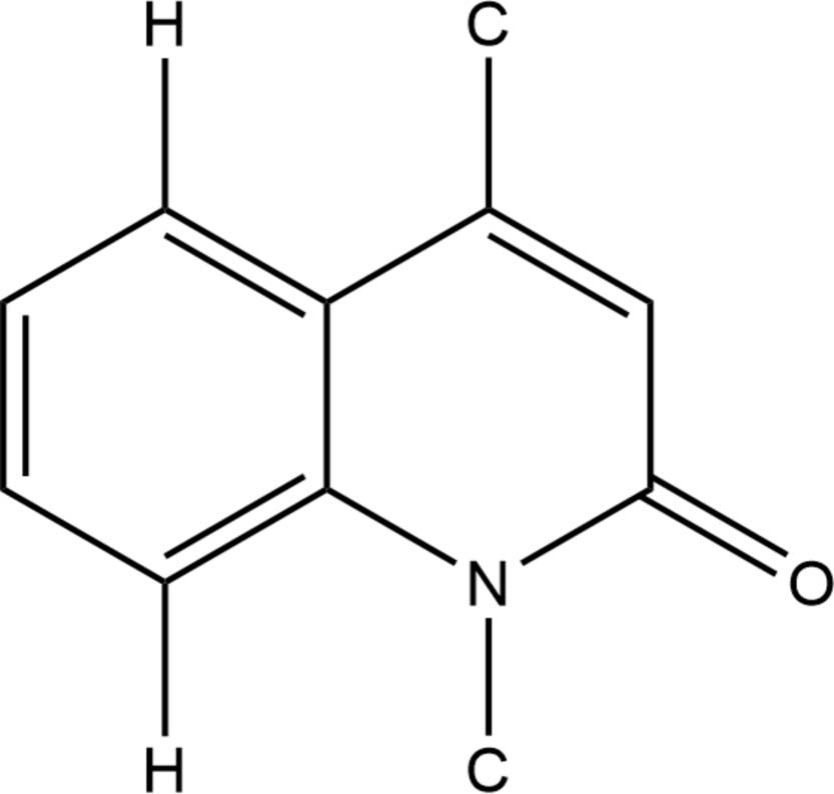


## Synthesis and crystallization

To a solution of 6-chloro-2-oxo-1,2-di­hydro­quinoline-4-carb­oxy­lic acid (1 g, 4.47 mmol) in 10 ml of DMF were added 3.30 ml (9.83 mmol) of methyl iodide, 3.17 g (22.36 mmol) of K_2_CO_3_ and 0.17 g (0.5 mmol) of tetra *n*-butyl­ammonium bromide (TBAB). The reaction mixture was stirred at room temperature in DMF for 6 h. After removal of salts, the solvent was evaporated under reduced pressure and the residue obtained was dissolved in di­chloro­methane. The organic phase was dried over Na_2_SO_4_ and then concentrated *in vacuo*. A pure compound was obtained after recrystallization from di­chloro­methane/hexane (*v*/*v* 1/3).

## Refinement

Crystal, data collection and refinement details are presented in Table 5 ▸. Hydrogen atoms were included as riding contributions in idealized positions with isotropic displacement parameters tied to those of the attached atoms. Two reflections obscured by the beamstop were omitted from the final refinement.

## Supplementary Material

Crystal structure: contains datablock(s) I, global. DOI: 10.1107/S2056989022002912/wm5635sup1.cif

Structure factors: contains datablock(s) I. DOI: 10.1107/S2056989022002912/wm5635Isup3.hkl

Supporting information file. DOI: 10.1107/S2056989022002912/wm5635Isup4.cdx

the infrared spectrum of b3lyp/6-311g of the compound (I). DOI: 10.1107/S2056989022002912/wm5635sup5.tif

Supporting information file. DOI: 10.1107/S2056989022002912/wm5635Isup5.cml

CCDC reference: 2159047

Additional supporting information:  crystallographic information; 3D view; checkCIF report

## Figures and Tables

**Table 1:** Hydrogen-bond geometry (Å, °)

*D*—H⋯*A*	*D*—H	H⋯*A*	*D*⋯*A*	*D*—H⋯*A*
C2—H2⋯O2^iii^	0.95	2.57	3.5146 (16)	178
C5—H5⋯O2	0.95	2.19	2.8496 (16)	126
C8—H8⋯Cl1^iv^	0.95	2.84	3.7786 (13)	170
C12—H12*C*⋯O1^ii^	0.98	2.36	3.0016 (16)	122

Symmetry codes: (ii) 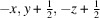 ; (iii) 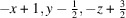 ; (iv) 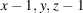 .

**Table 2:** Selected interatomic distances (Å)

Cl1⋯O3^i^	3.1903 (10)	H2⋯O2^iii^	2.57
Cl1⋯H8^i^	2.84	O3⋯H8	2.25
C12⋯O1^ii^	3.0016 (16)	C2⋯H10*C*	2.78
O2⋯C5	2.8496 (17)	C2⋯H10*B*	2.74
O1⋯H10*A*	2.26	C10⋯H2	2.46
H12*C*⋯O1^ii^	2.36	C11⋯H5	2.71
O2⋯H12*A*	2.64	H2⋯H10*B*	2.24
O2⋯H5	2.19	H2⋯H10*C*	2.29
O2⋯H12*B*	2.55		

Symmetry codes: (i) 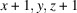 ; (ii) 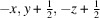 ; (iii) 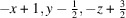 .

**Table 3:** B3LY/6–311G(d,p) equilibrium structural parameters (Å, °) and X-ray analysis of the title compound, (I)

Bonds/angles	X-ray	B3LYP/6–311G(d,p)
C2—C1	1.4070 (17)	1.4562
C3—C2	1.3547 (18)	1.3547
C4—C3	1.3836 (17)	1.3933
C5—C4	1.3807 (17)	1.3798
C6—C5	1.4098 (16)	1.4086
C7—C6	1.4514 (16)	1.4562
C8—C7	1.3510 (16)	1.3547
C9—C8	1.4513 (17)	1.4563
N1—C1	1.3920 (15)	1.3914
C10—N1	1.4673 (16)	1.4641
H2—C2	0.95	1.07965
H3—C3	0.95	1.0822
Cl1—C4	1.7397 (13)	1.7594
H5—C5	0.9500	1.0777
C11—C7	1.5022 (16)	1.5077
H8—C8	0.9500	1.0792
O1—C9	1.2319 (15)	1.2227
O2—C11	1.2040 (16)	1.2086
O3—C11	1.3258 (15)	1.3452
C12—O3	1.4458 (15)	1.4400
H12*A*—C12	0.98	1.0907
H12*B*—C12	0.98	1.0907
H12*C*—C12	0.98	1.0872
H10*A*—C10	0.98	1.0924
H10*B*—C10	0.98	1.0859
H10*C*—C10	0.98	1.0924
		
C3—C2—C1	120.15 (11)	121.15
C4—C3—C2	120.29 (12)	119.41
C5—C4—C3	121.22 (12)	121.07
C6—C5—C4	120.00 (11)	120.57
C7—C6—C5	123.66 (11)	123.69
C8—C7—C6	119.52 (11)	119.54
C9—C8—C7	123.22 (11)	123.86
N1—C1—C6	120.32 (11)	120.78
C10—N1—C1	119.77 (10)	120.37
H2—C2—C1	119.9	120.30
H3—C3—C4	119.9	120.32
Cl1—C4—C5	118.82 (10)	119.62
H5—C5—C6	120.00	119.03
C11—C7—C6	122.02 (10)	122.01
H8—C8—C9	118.40	114.79
O1—C9—N1	122.17 (12)	122.09
O2—C11—C7	125.71 (11)	125.86
O3—C11—C7	111.59 (10)	111.75
C12—O3—C11	116.08 (11)	115.75
H12*A*—C12—O3	109.5	110.40
H12*B*—C12—O3	109.5	110.40
H12*C*—C12—O3	109.5	105.31
H10*A*—C10—N1	109.5	110.62
H10*B*—C10—N1	109.5	107.00
H10*C*—C10—N1	109.5	110.40
		
C4—C3—C2—C1	0.40 (19)	0.00
C5—C4—C3—C2	0.32 (19)	0.00
C6—C5—C4—C3	−0.25 (19)	0.00
C7—C6—C5—C4	−179.83 (11)	−180.00
C8—C7—C6—C1	−1.59 (17)	0.00
C9—C8—C7—C6	0.23 (18)	0.00
N1—C1—C6—C7	0.69 (17)	0.00
C10—N1—C1—C6	−178.45 (12)	−180.0
Cl1—C4—C5—C6	−179.81 (9)	−180.0
O1—C9—N1—C1	177.48 (12)	179.99
O2—C11—C7—C6	−4.6 (2)	−0.01
O3—C11—C7—C6	175.14 (11)	−179.99
C12—O3—C11—C7	−179.85 (11)	179.99

**Table 4:** Calculated energies

Mol­ecular Energy (eV)	Compound (I)
Total Energy *TE* (eV)	−32759.86
*E*_HOMO_ (eV)	−6.50
*E*_LUMO_ (eV)	−2.82
Gap, *ΔE* (eV)	3.68
Dipole moment, *μ* (Debye)	0.6065
Ionization potential, *I* (eV)	6.50
Electron affinity, *A*	2.82
Electronegativity, *χ*	1.84
Hardness, *η*	3.68
Softness, *σ*	0.27
Electrophilicity index, *ω*	−0.68

**Table 5:** Experimental details

Crystal data	
Chemical formula	C_12_H_10_ClNO_3_
*M*_r_	251.66
Crystal system, space group	Monoclinic, *P*2_1_/*c*
Temperature (K)	150
*a*, *b*, *c* (Å)	8.3515 (3), 16.6672 (6), 7.9705 (3)
β (°)	107.191 (2)
*V* (Å^3^)	1059.90 (7)
*Z*	4
Radiation type	Mo *K*α
μ (mm^−1^)	0.36
Crystal size (mm)	0.35 × 0.19 × 0.04

Data collection	
Diffractometer	Bruker D8 QUEST PHOTON 3 diffractometer
Absorption correction	Multi-scan (*SADABS*; Krause *et al.*, 2015 ▸)
*T*_min_, *T*_max_	0.94, 0.99
No. of measured, independent and observed [*I* > 2σ(*I*)] reflections	70718, 3544, 2977
*R*_int_	0.040
(sin θ/λ)_max_ (Å^−1^)	0.737

Refinement	
*R*[*F* ^2^ > 2σ(*F* ^2^)], *wR*(*F* ^2^), *S*	0.041, 0.115, 1.06
No. of reflections	3544
No. of parameters	156
H-atom treatment	H-atom parameters constrained
Δρ_max_, Δρ_min_ (e Å^−3^)	0.54, −0.30

Computer programs: *APEX3* and *SAINT* (Bruker, 2020 ▸), *SHELXT* (Sheldrick, 2015*a* ▸), *SHELXL* (Sheldrick, 2015*b* ▸), *DIAMOND* (Brandenburg & Putz, 2012 ▸) and *publCIF* (Westrip, 2010 ▸).

## supplementary crystallographic information

### Crystal data

**Table:**

C_12_H_10_ClNO_3_	*F*(000) = 520
*M**_r_* = 251.66	*D*_x_ = 1.577 Mg m^−^^3^
Monoclinic, *P*2_1_/*c*	Mo *K*α radiation, λ = 0.71073 Å
*a* = 8.3515 (3) Å	Cell parameters from 9727 reflections
*b* = 16.6672 (6) Å	θ = 2.6–31.5°
*c* = 7.9705 (3) Å	µ = 0.36 mm^−^^1^
β = 107.191 (2)°	*T* = 150 K
*V* = 1059.90 (7) Å^3^	Plate, pale blue
*Z* = 4	0.35 × 0.19 × 0.04 mm

### Data collection

**Table:**

Bruker D8 QUEST PHOTON 3 diffractometer	3544 independent reflections
Radiation source: fine-focus sealed tube	2977 reflections with *I* > 2σ(*I*)
Graphite monochromator	*R*_int_ = 0.040
Detector resolution: 7.3910 pixels mm^-1^	θ_max_ = 31.6°, θ_min_ = 2.8°
φ and ω scans	*h* = −12→12
Absorption correction: multi-scan (*SADABS*; Krause *et al.*, 2015)	*k* = −24→24
*T*_min_ = 0.94, *T*_max_ = 0.99	*l* = −11→11
70718 measured reflections	

### Refinement

**Table:**

Refinement on *F*^2^	Primary atom site location: dual
Least-squares matrix: full	Secondary atom site location: difference Fourier map
*R*[*F*^2^ > 2σ(*F*^2^)] = 0.041	Hydrogen site location: inferred from neighbouring sites
*wR*(*F*^2^) = 0.115	H-atom parameters constrained
*S* = 1.06	*w* = 1/[σ^2^(*F*_o_^2^) + (0.0614*P*)^2^ + 0.427*P*] where *P* = (*F*_o_^2^ + 2*F*_c_^2^)/3
3544 reflections	(Δ/σ)_max_ = 0.001
156 parameters	Δρ_max_ = 0.54 e Å^−^^3^
0 restraints	Δρ_min_ = −0.30 e Å^−^^3^

### Special details

**Table:**

Experimental. The diffraction data were obtained from 12 sets of frames, each of width 0.5° in ω or φ, collected with scan parameters determined by the "strategy" routine in *APEX3*. The scan time was 15 sec/frame.
Geometry. All esds (except the esd in the dihedral angle between two l.s. planes) are estimated using the full covariance matrix. The cell esds are taken into account individually in the estimation of esds in distances, angles and torsion angles; correlations between esds in cell parameters are only used when they are defined by crystal symmetry. An approximate (isotropic) treatment of cell esds is used for estimating esds involving l.s. planes.
Refinement. Refinement of F^2^ against ALL reflections. The weighted R-factor wR and goodness of fit S are based on F^2^, conventional R-factors R are based on F, with F set to zero for negative F^2^. The threshold expression of F^2^ > 2sigma(F^2^) is used only for calculating R-factors(gt) etc. and is not relevant to the choice of reflections for refinement. R-factors based on F^2^ are statistically about twice as large as those based on F, and R- factors based on ALL data will be even larger. H-atoms attached to carbon were placed in calculated positions (C—H = 0.95 - 0.98 Å). All were included as riding contributions with isotropic displacement parameters 1.2 - 1.5 times those of the attached atoms. Two reflections obscured by the beamstop were omitted from the final refinement.

### Fractional atomic coordinates and isotropic or equivalent isotropic displacement parameters (Å^2^)

**Table:**

	*x*	*y*	*z*	*U*_iso_*/*U*_eq_	
Cl1	0.84517 (4)	0.57446 (2)	1.07652 (4)	0.02841 (10)	
O1	0.11119 (15)	0.35446 (6)	0.34782 (14)	0.0330 (2)	
O2	0.33909 (15)	0.67466 (6)	0.64348 (17)	0.0415 (3)	
O3	0.12761 (13)	0.64014 (6)	0.41182 (12)	0.0279 (2)	
N1	0.34559 (13)	0.36878 (6)	0.58343 (13)	0.0213 (2)	
C1	0.46120 (15)	0.41783 (7)	0.70040 (16)	0.0199 (2)	
C2	0.59973 (16)	0.38341 (8)	0.82548 (17)	0.0236 (2)	
H2	0.612806	0.326763	0.831409	0.028*	
C3	0.71602 (16)	0.43149 (7)	0.93894 (17)	0.0226 (2)	
H3	0.809737	0.407995	1.022678	0.027*	
C4	0.69735 (16)	0.51402 (8)	0.93189 (16)	0.0223 (2)	
C5	0.56211 (15)	0.54992 (7)	0.81228 (16)	0.0205 (2)	
H5	0.551223	0.606682	0.809742	0.025*	
C6	0.44019 (14)	0.50236 (7)	0.69390 (15)	0.0187 (2)	
C7	0.29453 (15)	0.53504 (7)	0.56374 (15)	0.0186 (2)	
C8	0.18725 (16)	0.48494 (7)	0.45164 (16)	0.0213 (2)	
H8	0.092808	0.507382	0.367006	0.026*	
C9	0.20920 (16)	0.39853 (8)	0.45415 (16)	0.0228 (2)	
C10	0.36647 (19)	0.28134 (8)	0.59449 (19)	0.0288 (3)	
H10A	0.272556	0.255765	0.506926	0.043*	
H10B	0.369000	0.263150	0.712165	0.043*	
H10C	0.471808	0.266706	0.571760	0.043*	
C11	0.25956 (15)	0.62357 (7)	0.54768 (16)	0.0209 (2)	
C12	0.08323 (19)	0.72398 (8)	0.3841 (2)	0.0304 (3)	
H12A	0.174601	0.753257	0.357605	0.046*	
H12B	0.064284	0.746230	0.490485	0.046*	
H12C	−0.019260	0.729287	0.285546	0.046*	

### Atomic displacement parameters (Å^2^)

**Table:**

	*U*^11^	*U*^22^	*U*^33^	*U*^12^	*U*^13^	*U*^23^
Cl1	0.02388 (16)	0.02942 (17)	0.02545 (16)	−0.00297 (11)	−0.00271 (12)	−0.00145 (11)
O1	0.0406 (6)	0.0229 (5)	0.0279 (5)	−0.0084 (4)	−0.0016 (4)	−0.0034 (4)
O2	0.0370 (6)	0.0184 (4)	0.0512 (7)	0.0009 (4)	−0.0147 (5)	−0.0053 (4)
O3	0.0309 (5)	0.0208 (4)	0.0250 (5)	0.0054 (4)	−0.0028 (4)	0.0003 (3)
N1	0.0261 (5)	0.0153 (4)	0.0205 (5)	0.0001 (4)	0.0041 (4)	−0.0008 (3)
C1	0.0217 (5)	0.0175 (5)	0.0201 (5)	−0.0002 (4)	0.0056 (4)	−0.0002 (4)
C2	0.0249 (6)	0.0198 (5)	0.0251 (6)	0.0033 (4)	0.0061 (5)	0.0024 (4)
C3	0.0222 (6)	0.0223 (5)	0.0222 (5)	0.0027 (4)	0.0048 (4)	0.0022 (4)
C4	0.0202 (5)	0.0242 (5)	0.0200 (5)	−0.0009 (4)	0.0023 (4)	−0.0003 (4)
C5	0.0205 (5)	0.0186 (5)	0.0210 (5)	0.0002 (4)	0.0039 (4)	0.0001 (4)
C6	0.0189 (5)	0.0178 (5)	0.0182 (5)	0.0002 (4)	0.0035 (4)	−0.0002 (4)
C7	0.0196 (5)	0.0162 (5)	0.0188 (5)	0.0000 (4)	0.0041 (4)	0.0002 (4)
C8	0.0221 (5)	0.0188 (5)	0.0206 (5)	−0.0012 (4)	0.0027 (4)	0.0004 (4)
C9	0.0262 (6)	0.0202 (5)	0.0203 (5)	−0.0025 (4)	0.0043 (4)	0.0001 (4)
C10	0.0364 (7)	0.0153 (5)	0.0323 (7)	0.0006 (5)	0.0063 (6)	0.0007 (4)
C11	0.0206 (5)	0.0176 (5)	0.0221 (5)	0.0008 (4)	0.0027 (4)	0.0008 (4)
C12	0.0334 (7)	0.0221 (6)	0.0314 (7)	0.0077 (5)	0.0030 (5)	0.0055 (5)

### Geometric parameters (Å, º)

**Table:**

Cl1—C4	1.7397 (13)	C4—C5	1.3807 (17)
O1—C9	1.2319 (15)	C5—C6	1.4098 (16)
O2—C11	1.2040 (16)	C5—H5	0.9500
O3—C11	1.3258 (15)	C6—C7	1.4514 (16)
O3—C12	1.4458 (15)	C7—C8	1.3510 (16)
N1—C9	1.3825 (16)	C7—C11	1.5022 (16)
N1—C1	1.3920 (15)	C8—C9	1.4513 (17)
N1—C10	1.4673 (16)	C8—H8	0.9500
C1—C2	1.4070 (17)	C10—H10A	0.9800
C1—C6	1.4189 (16)	C10—H10B	0.9800
C2—C3	1.3726 (18)	C10—H10C	0.9800
C2—H2	0.9500	C12—H12A	0.9800
C3—C4	1.3836 (17)	C12—H12B	0.9800
C3—H3	0.9500	C12—H12C	0.9800
			
Cl1···O3^i^	3.1903 (10)	H2···O2^iii^	2.57
Cl1···H8^i^	2.84	O3···H8	2.25
C12···O1^ii^	3.0016 (16)	C2···H10C	2.78
O2···C5	2.8496 (17)	C2···H10B	2.74
O1···H10A	2.26	C10···H2	2.46
H12C···O1^ii^	2.36	C11···H5	2.71
O2···H12A	2.64	H2···H10B	2.24
O2···H5	2.19	H2···H10C	2.29
O2···H12B	2.55		
			
C11—O3—C12	116.08 (11)	C8—C7—C11	118.46 (11)
C9—N1—C1	122.97 (10)	C6—C7—C11	122.02 (10)
C9—N1—C10	117.27 (10)	C7—C8—C9	123.22 (11)
C1—N1—C10	119.77 (10)	C7—C8—H8	118.4
N1—C1—C2	119.83 (11)	C9—C8—H8	118.4
N1—C1—C6	120.32 (11)	O1—C9—N1	122.17 (12)
C2—C1—C6	119.85 (11)	O1—C9—C8	121.78 (12)
C3—C2—C1	120.15 (11)	N1—C9—C8	116.05 (11)
C3—C2—H2	119.9	N1—C10—H10A	109.5
C1—C2—H2	119.9	N1—C10—H10B	109.5
C2—C3—C4	120.29 (12)	H10A—C10—H10B	109.5
C2—C3—H3	119.9	N1—C10—H10C	109.5
C4—C3—H3	119.9	H10A—C10—H10C	109.5
C5—C4—C3	121.22 (12)	H10B—C10—H10C	109.5
C5—C4—Cl1	118.82 (10)	O2—C11—O3	122.70 (12)
C3—C4—Cl1	119.96 (10)	O2—C11—C7	125.71 (11)
C4—C5—C6	120.00 (11)	O3—C11—C7	111.59 (10)
C4—C5—H5	120.0	O3—C12—H12A	109.5
C6—C5—H5	120.0	O3—C12—H12B	109.5
C5—C6—C1	118.48 (11)	H12A—C12—H12B	109.5
C5—C6—C7	123.66 (11)	O3—C12—H12C	109.5
C1—C6—C7	117.86 (10)	H12A—C12—H12C	109.5
C8—C7—C6	119.52 (11)	H12B—C12—H12C	109.5
			
C9—N1—C1—C2	−178.19 (12)	C1—C6—C7—C8	−1.59 (17)
C10—N1—C1—C2	1.67 (18)	C5—C6—C7—C11	−1.54 (18)
C9—N1—C1—C6	1.70 (18)	C1—C6—C7—C11	179.15 (11)
C10—N1—C1—C6	−178.45 (12)	C6—C7—C8—C9	0.23 (18)
N1—C1—C2—C3	178.70 (11)	C11—C7—C8—C9	179.51 (11)
C6—C1—C2—C3	−1.18 (19)	C1—N1—C9—O1	177.48 (12)
C1—C2—C3—C4	0.40 (19)	C10—N1—C9—O1	−2.39 (19)
C2—C3—C4—C5	0.32 (19)	C1—N1—C9—C8	−2.97 (17)
C2—C3—C4—Cl1	179.88 (10)	C10—N1—C9—C8	177.17 (11)
C3—C4—C5—C6	−0.25 (19)	C7—C8—C9—O1	−178.44 (13)
Cl1—C4—C5—C6	−179.81 (9)	C7—C8—C9—N1	2.01 (18)
C4—C5—C6—C1	−0.52 (18)	C12—O3—C11—O2	−0.1 (2)
C4—C5—C6—C7	−179.83 (11)	C12—O3—C11—C7	−179.85 (11)
N1—C1—C6—C5	−178.65 (11)	C8—C7—C11—O2	176.09 (14)
C2—C1—C6—C5	1.23 (18)	C6—C7—C11—O2	−4.6 (2)
N1—C1—C6—C7	0.69 (17)	C8—C7—C11—O3	−4.12 (16)
C2—C1—C6—C7	−179.43 (11)	C6—C7—C11—O3	175.14 (11)
C5—C6—C7—C8	177.71 (12)		

Symmetry codes: (i) *x*+1, *y*, *z*+1; (ii) −*x*, *y*+1/2, −*z*+1/2; (iii) −*x*+1, *y*−1/2, −*z*+3/2.

### Hydrogen-bond geometry (Å, º)

**Table:**

*D*—H···*A*	*D*—H	H···*A*	*D*···*A*	*D*—H···*A*
C2—H2···O2^iii^	0.95	2.57	3.5146 (16)	178
C5—H5···O2	0.95	2.19	2.8496 (16)	126
C8—H8···Cl1^iv^	0.95	2.84	3.7786 (13)	170
C12—H12*C*···O1^ii^	0.98	2.36	3.0016 (16)	122

Symmetry codes: (ii) −*x*, *y*+1/2, −*z*+1/2; (iii) −*x*+1, *y*−1/2, −*z*+3/2; (iv) *x*−1, *y*, *z*−1.
