# Supplementary figures and images for: Crystal structure, Hirshfeld surface analysis, inter­action energy and DFT calculations and energy frameworks of methyl 6-chloro-1-methyl-2-oxo-1,2-di­hydro­quinoline-4-carboxyl­ate
Source: Acta Crystallogr E Crystallogr Commun. 2022 Mar 22;78(Pt 4):425–32. doi: 10.1107/S2056989022002912 (PMC8983989; doi:10.1107/S2056989022002912)

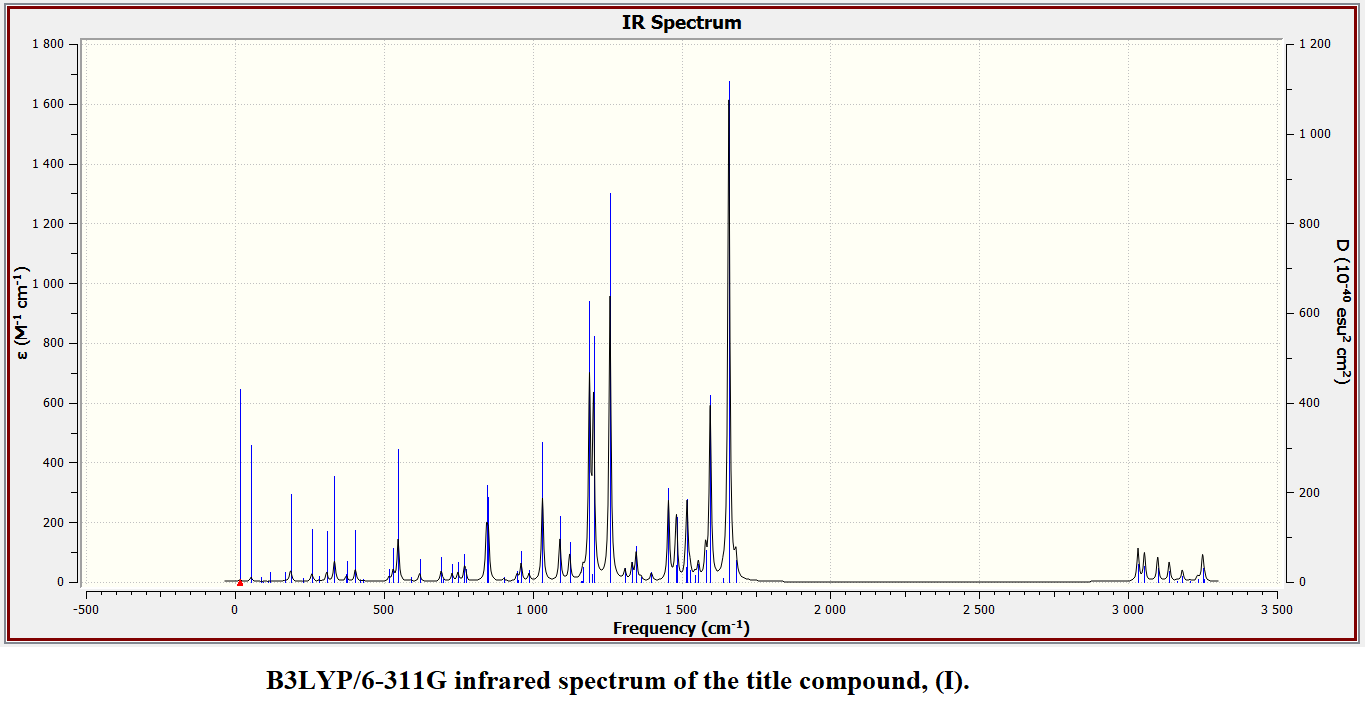

Supplement: Supplementary file 4 [file e-78-00425-sup5.tif]
